# Robotics-Assisted Pediatric Oncology Surgery—A Preliminary Single-Center Report and a Systematic Review of Published Studies

**DOI:** 10.3389/fped.2021.780830

**Published:** 2022-01-18

**Authors:** Fabrizio Vatta, Marta Gazzaneo, Mirko Bertozzi, Alessandro Raffaele, Luigi Avolio, Giovanna Riccipetitoni

**Affiliations:** Department of Pediatric Surgery, Fondazione IRCCS Policlinico San Matteo, Pavia, Italy

**Keywords:** robotic-assisted surgery, oncology, pediatrics, children, mini-invasive surgery

## Abstract

**Aim:**

The use of robotics-assisted surgery in oncology has been proved effective and safe in adults. Despite these results, the use of robotics has been rarely reported for pediatric oncology. Our review aims to evaluate the safety and feasibility of robotics-assisted surgery in this field, analyzing our experience and performing a systematic review of the most recent studies.

**Methods:**

We reviewed all patients affected by an oncological disease who underwent a robotics-assisted procedure at our institute. We performed a systematic review of the literature from 2012 to 2021 on the subjects.

**Findings:**

A total of 14 patients underwent robotics-assisted tumor resection. Eleven procedures (median age 13.2-years old) were carried out in children with adnexal lesions (seven tumor excision and four ovariectomies). Histological diagnosis was mature teratoma (six), serous papillary cystadenofibromas of the fallopian tube (two), ovarian serous cystadenoma (one), ovarian mucinous cystadenoma (one), and ovarian seromucinous cystadenoma. The median length of stay was 2 days. No recurrences or complications at a median follow-up of 2.1-years were observed. A 5-year-old girl underwent a complete posterior resection of a type 3 sacrococcygeal tumor with a robotics-assisted approach for the dissection of a possible intraabdominal residual component of the lesion. No intra- and postoperative complications were recorded. Complete excision of a recurrent differentiating neuroblastoma of the left para-renal region was performed on a 9-year-old girl. An idiopathic anaphylactic shock occurred 1 day after the procedure. At 9 months' follow-up, no local recurrences of the lesion were observed. Overall, we reported no conversion to open surgery. Lastly, a robotic excision of a growing left superior mediastinal intermixed ganglioneuroblastoma was performed on an 8-year-old girl with no postoperative complications. Follow-up was uneventful (7 months). In the literature, the rate of complications ranges from 0 to 28%, mainly related to difficult dissection and impaired anatomy. Conversion is reported in 5% of all oncological procedures, due to more invading tumors and altered anatomical features. No robotics-related complications were reported.

**Conclusion:**

Robotics-assisted surgery in pediatric oncology has proven to be feasible. Nevertheless, its use should be limited to selected cases and performed by highly trained oncological surgeons. Preparation and patient positioning, alongside a correct port placement, are crucial to carrying out these procedures. Further innovations in robotics may allow a wider application of this technology in pediatric oncology.

## Introduction

Robotics-assisted surgery (RAS) represents one of the most important advancements in minimally invasive surgery (MIS) in recent years and has progressively gained a predominant role in many fields of adult surgery. The da Vinci surgical system (Intuitive Surgical, Inc., Sunnyvale, CA, USA) is actually present in 67 countries, and more than 5,500 robots are used worldwide (1).

Well-known advantages are a stable magnified 3D view, tremor filtering, and motion scaling, which allow precise intracorporeal exposure and suturing (2).

RAS in pediatric surgery has initially struggled due to some limitations, notably port and arm dimensions, as well as high costs (3). Nevertheless, the growing number of case reports and series published every year reveals how RAS is increasingly applied for children (4).

Despite this spread, its use for pediatric oncology is still limited, and few studies have been conducted on the subject. The reasons are represented by the characteristics of pediatric tumors, as each type may be considered a rare disease. Moreover, most pediatric malignancies are embryonal tumors with rapid growth, which require frequently other therapies as neoadjuvant chemotherapy. All these distinctive features limit the creation of guidelines for the robotic approach. Nonetheless, accepted recommendations require an evaluation by a multidisciplinary tumor board and respecting oncological protocols for open surgery for each specific pathology (5).

We performed a retrospective study to critically review our experience in RAS.

In order to compare our results with those from the literature, we performed a systematic review, focusing on technical skills that could help pediatric surgeons to avoid intra- and postoperative complications.

## Materials and Methods

We performed a retrospective review of all pediatric oncological patients who underwent RAS at our institution from 2010 to 2021. From 2017 to 2020, the use of the robotic platform has been suspended due to technical reasons.

Patients over 18-years old were excluded, as well as all malignancies not treated with RAS.

We analyzed demographic data, including age at surgery, sex, pathology, possible comorbidities, operation time (OT), length of hospital stay (LHS), perioperative complications, and postoperative outcomes. Postoperative complications were classified according to the Clavien–Dindo classification and graded from I to V.

All procedures were carried out using the da Vinci Si Surgical System (Intuitive Surgical, Sunnyvale, CA, USA). All surgeries were performed with three robotic arms, placed accordingly depending on the lesion site and size. Some procedures required an accessory port (3 or 5 mm).

To compare our results with those of the literature, a systematic review was performed according to the Preferred Reporting Items for Systematic Reviews and Meta-Analyses (PRISMA) criteria.

We selected articles reporting RAS in oncological pediatric patients between 2012 and September 2021 in MEDLINE and EMBASE using the following keywords: “(pediatric) or (children) and (robot) or (robotic) and (oncology) or (tumor).”

Inclusion criteria were as follows:

- Articles published between January 2012 and September 2021
- Articles written in English
- Median/mean age <18-years
- Case series with more than 3 patients
- Articles where data concerning demographics, surgical indications, complication, and conversion rates were clearly deductible.

All data were elaborated using the statistical software “R,” version 3.4.1. Descriptive statistics were used to present findings, and quantitative variables were expressed as median (range) to express our data. Data elaborated from the literature review were expressed as median (range) or mean ± SDs depending on the reference found in the original articles.

## Results

A total of 14 pediatric patients underwent RAS for oncological pathologies from 2010 to 2021 at our institute. All data are displayed in Table 1. No patients required a conversion to open surgery.

**Table 1:** Summarized data of all oncological patients undergoing RAS (in chronological order).

**Year**	**Patient**	**Sex**	**Age**	**Region/specialty**	**Surgical intervention**	**Side**	**Diagnosis**	**Robotic port (optic–operative–operative)**	**Accessory port**	**OT**	**Conversion**	**Perioperative complication**	**Postoperative complication**	**LHS (days)**	**Follow-up (years)**
2011	NA	F	8.6	Gynecology	Ovariectomy	L	Ovarian mature cystic teratoma	8–5–5	No	130	No	No	No	1	0.63
2011	PLC	F	13.2	Gynecology	Ovariectomy	L	Ovarian mature cystic teratoma	8–5–5	No	260	No	No	No	2	6.11
2012	BG	F	5.4	Abdomen	Robotics-assisted explorative laparoscopy (mass debulking via posterior approach)	NA	Mature sacrococcygeal teratoma	8–5–5	No	NA	No	No	No	4	LAF
2015	AS	F	8.7	Gynecology	Ovariectomy	L	Ovarian mature cystic teratoma	8–8–8	1 (3 mm)	215	No	No	No	2	6.29
2015	KA	F	14.8	Gynecology	Mass excision	R	Ovarian seromucinous cystadenoma	8–5–5	No	120	No	Spillage	No	2	5.58
2016	SS	F	12.9	Gynecology	Mass excision	L	Serous papillary cystadenofibroma of the fallopian tube	8–5–5	No	105	No	No	No	1	0.88
2017	BN	F	16.9	Gynecology	Ovariectomy	R	Ovarian mature cystic teratoma	12–8–8	No	195	No	No	No	2	3.28
2017	GC	F	8.0	Gynecology	Mass excision	L	Ovarian mucinous cystadenoma	8–5–5	No	65	No	No	No	1	0.15
2017	CSE	F	13.6	Gynecology	Mass excision (concomitant urachal remnant excision)	L	Ovarian serous cystadenoma	8–5–5	No	155	No	No	No	4	3.32
2017	CV	F	16.4	Gynecology	Mass excision	R	Serous papillary cystadenofibroma of the fallopian tube	8–5–5	No	90	No	No	No	2	0.92
2020	GA	F	9.4	Abdomen	Mass excision	L	Differentiating neuroblastoma	12–8–8	1 (5 mm)	320	No	No	Anaphylactic shock (1 day postop)—Cl. Dindo IV	8	1.22^*^
2020	FE	F	7.6	Thoracic	Mass excision	L	Intermixed ganglioneuroblastoma	8–8–8	1 (5 mm)	290	No	No	No	7	0.78
2020	SM	F	13.5	Gynecology	Mass excision	R	Ovarian mature cystic teratoma	8–5–5	1 (5 mm)	110	No	No	No	2	LAF
2021	SG	F	13.1	Gynecology	Mass excision	L	Ovarian mature cystic teratoma	12–8–8	1 (5 mm)	105	No	Spillage	No	2	0.63

*RAS, robotics-assisted surgery; OT, operation time; LHS, length of hospital stay*.

* *Neuroblastoma localization in a supraclavicular lymph node at 6 months postop treated surgically*.

**Figure 1:**
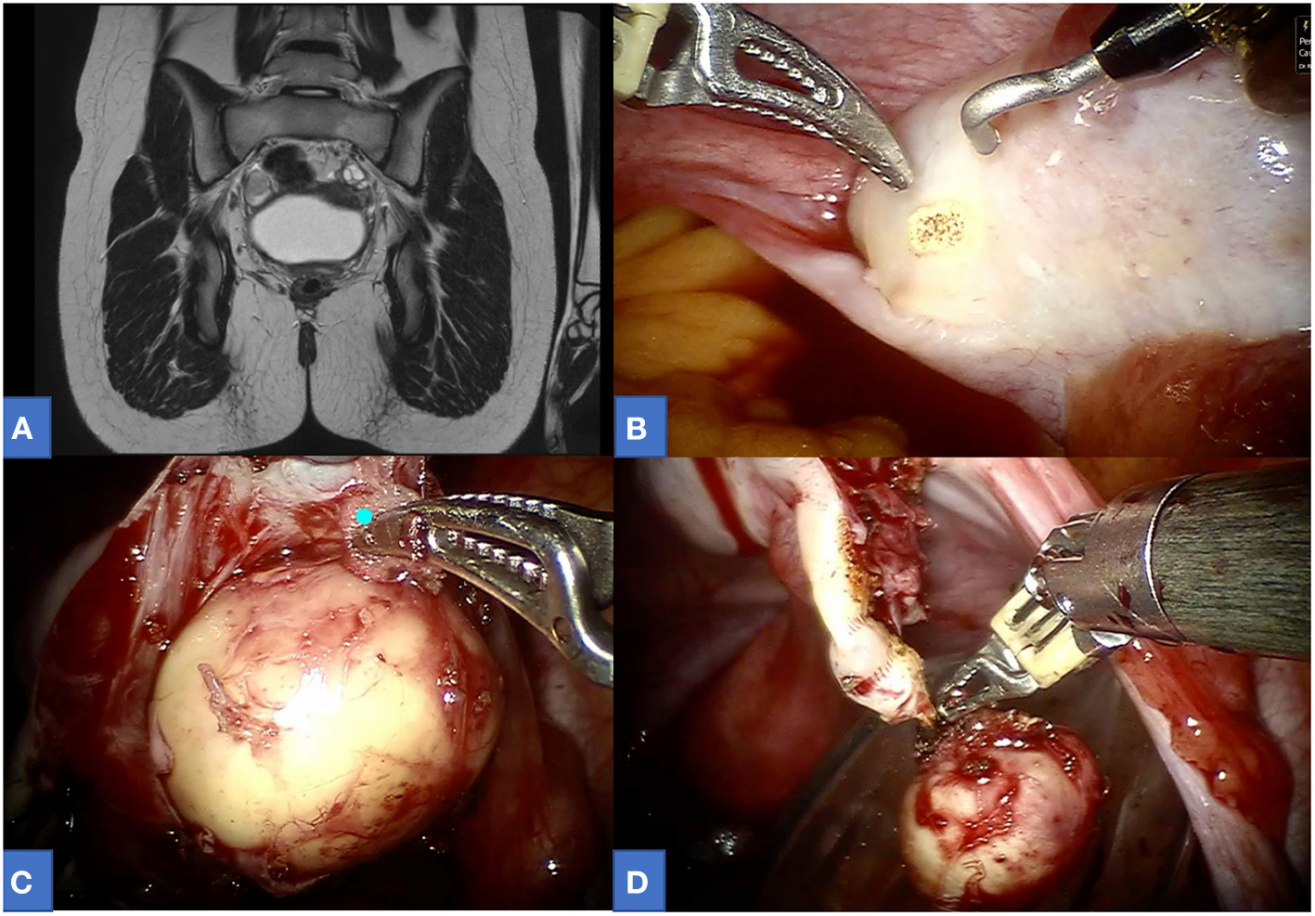
A 13-year-old affected by right ovarian mature teratoma. **(A)** Preoperative MRI. **(B–D)** Intraoperative view.

Among our cohort, 11 gynecological procedures were performed (7 mass excisions and 4 ovariectomies) for the following tumors: 6 ovarian mature cystic teratomas (Figure 1), 2 serous papillary cystadenofibroma of the fallopian tube, 1 ovarian mucinous cystadenoma, 1 ovarian serous cystadenoma, and 1 ovarian seromucinous cystadenoma. The median age at surgery was 13.2 [8.0–16.9], with median operative time including docking time 120 [65–260]. Most of the procedures were carried out using an 8-mm optic port and two 5-mm operative ports. In only three patients, an additional accessory port (either 5 or 3 mm) was positioned. In two cases (14.2%), intraoperative spillage was reported. No other perioperative complications nor conversion were reported. Follow-up was uneventful for all patients (median follow-up 2.1-years [0.2–6.3]). The median length of hospitalization was 2 days (1–4).

We performed one thoracic procedure on a 7-year-old girl for a growing intermixed ganglioneuroblastoma located on the supero-posterior mediastinum encasing the subclavian vessels. An 8-mm optic port was positioned in the sixth intercostal space on the midaxillary line. Two 8-mm operative ports were positioned 8 cm away from the optic port, in the fifth intercostal space on the anterior axillary line and in the seventh intercostal space on the paravertebral line. Finally, a 5-mm auxiliary port was placed in the fourth intercostal space on the anterior axillary line. No postoperative complications were reported.

We report one robotics-assisted explorative laparoscopy on a 5-year-old girl who previously underwent posterior excision of a type 3 mature sacrococcygeal teratoma, as the preoperative imaging showed suspicion of tumor extension in the pelvis. The robotics-assisted exploration result was negative.

We completed an excision of a left perirenal recurrence of neuroblastoma in a 9-year-old girl. A 12-mm optic port was placed trans-umbilically, whereas two 8-mm operative ports were placed in the left hypochondrium and in the left iliac region. An accessory port was then positioned in the epigastric region. No intraoperative complication occurred. The patient suffered from an anaphylactic shock on the first postoperative day that required adrenaline and corticosteroid administration. Further postoperative course was uneventful.

**Figure 2:**
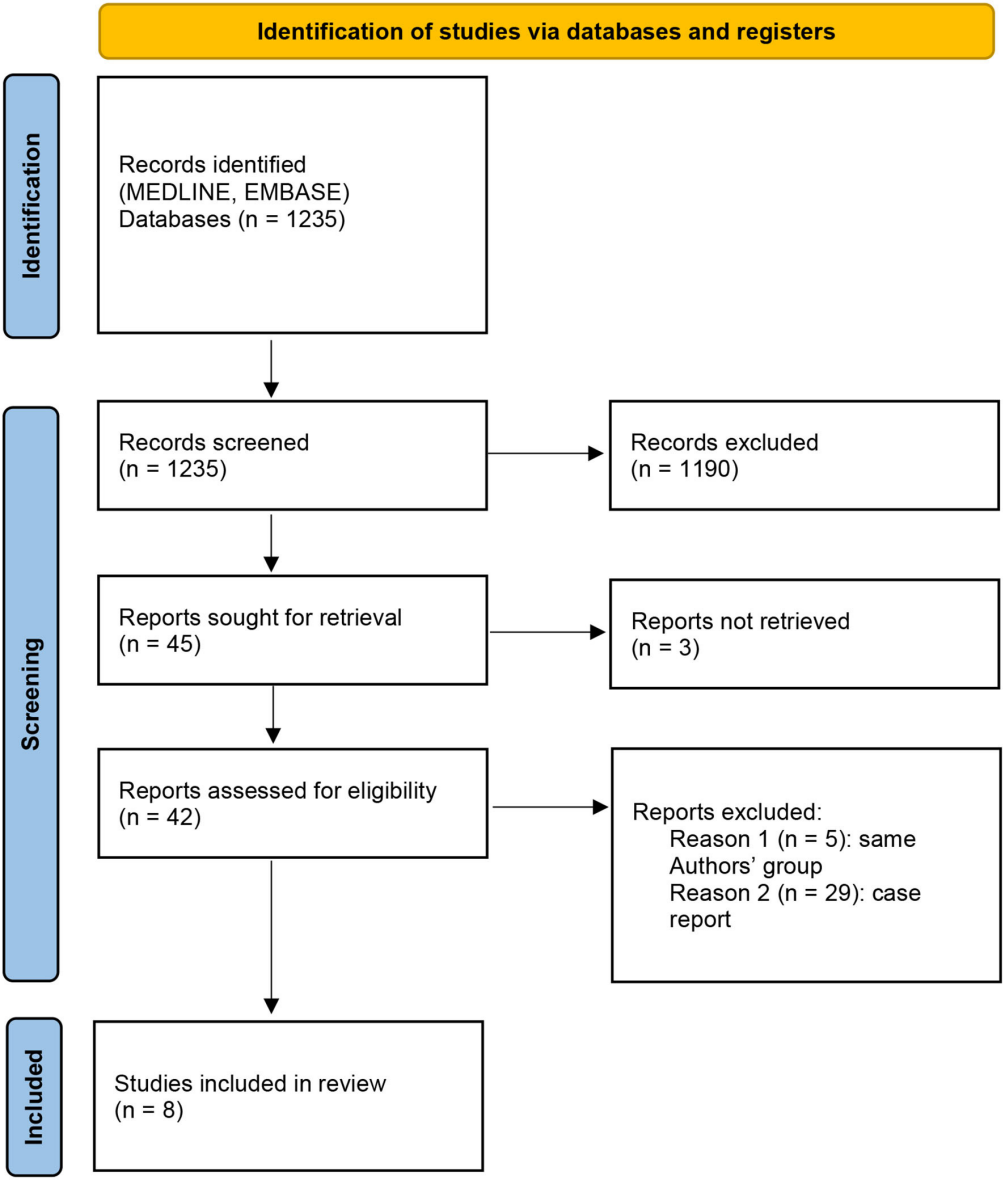
Preferred Reporting Items for Systematic Reviews and Meta-Analyses (PRISMA) 2020 flow diagram.

A systematic review was performed according to the PRISMA guidelines (Figure 2). Eight studies met the eligibility criteria, for a total of 137 procedures in 134 patients. Data are summarized in Table 2. The male-to-female (M:F) ratio was ~1:2, the median age was 9-years [0.9–19.0], and the median weight was 35 kg (when reported). Treated conditions were represented by a broad group of tumors, and the most common were adrenal. The malignancy rate was on average 65%. The median conversion to open surgery rate was 5%.

**Table 2:** Results of the systematic review.

**Author**	**Publication year**	**Patients (n)**	**Tumor type**	**Diagnosis**	**M:F ratio**	**Procedures (n)**	**Median age (years)**	**Median weight (kg)**	**Mean total operative time (minutes)**	**Malignancy rate (%)**	**Conversion rate (%)**	**Intraoperative complications (%)**	**Type of intraoperative complication/cause of conversion**	**Postoperative complication rate (%)**	**Clavien–Dindo complications**	**Postoperative complications**	**LHS (Days)**	**Follow-up (months)**	**Recurrence rate (%)**
Meehan (5)	2013	14	Mediastinal (*n =* 4), retroperitoneal (*n =* 4), adrenal (*n =* 3), ovarian (*n =* 1), colonic (*n =* 1), pancreatic (*n =* 1)	Germ cell mediastinal tumor (*n =* 1), mature mediastinal teratoma (*n =* 1), ganglioneuroma (*n =* 2), ganglioneuroblastoma (*n =* 1), neuroblastoma (*n =* 3), pheochromocytoma (*n =* 1), adrenal carcinoma (*n =* 1), begin adrenal mass (*n =* 1), colon cancer (*n =* 1), pancreatic tumor (*n =* 1)	NA	14	NA	NA	NA	NA	28 (*n =* 4)	28 (*n =* 4)	Not confident with the anatomy (*n =* 2: retroperitoneal ganglioneuroma, pancreatic mass), acute hypertensive crisis during adrenal pheochromocytoma resection (*n =* 1), unexpected discovery of a large colon tumor invading the anterior abdominal wall (*n =* 1)	0	/		NA	NA	0
Varda et al. (6)	2018	7	Renal (*n =* 4), retroperitoneal (*n =* 2), adrenal (*n =* 1)	Ganglioneuroma (*n =* 1), papillary renal cell carcinoma (*n =* 1), non-seminomatous germ cell tumors (*n =* 1), renal tumor ns (*n =* 1), rhabdomyosarcoma (*n =* 1), cystic renal dysplasia (*n =* 2)	NA	7	12.5 (3–19)	45 (14–79)	277 (172–508)	42 (*n =* 3)	0	0		0	/		NA	7	0
Xie et al. (7)	2019	4	Ovarian (*n =* 4)	Ovarian mature cystic teratoma (*n =* 2), mucinous tumor (*n =* 1), ovarian teratoma (*n =* 1)	0:04	4	7.5 (1–13)	36.8 (8.5 –69.5)	120	NA	0	0		0	/		3	6	0
Navarrete Arellano et al. (8)	2019	4	Mediastinal teratoma (n =), renal (*n =* 1), retroperitoneal (*n =* 1), adrenal (*n =* 1)	Mediastinal teratoma (*n =* 1), retroperitoneal lipoma (*n =* 1), pheochromocytoma (*n =* 1), renal tumor ns (*n =* 1)	NA	4	NA	NA	NA	NA	NA	NA		0	/		2.6	NA	NA
Esposito et al. (9)	2020	5	Ovarian (*n =* 5)	Mature teratoma (*n =* 3), seromucinous cystadenoma (*n =* 2)	0:05	5	13.5 (11–16)	NA	78 (66–90)	0	0	0		0	/		NA	NA	NA
Mitra et al. (10)	2020	3	Adrenal (*n =* 3)	Ganglioneuroblastoma (*n =* 2), pheochromocytoma (*n =* 1)	2:01	3	6.3 (2–13)	NA	244 (244–265)	NA	0	0		33 (*n =* 1)	II (*n =* 1)	Unexpected drug reaction (*n =* 1)	2	19 (12–30)	0
Blanc et al. (11)	2021	89	Neuroblastic (*n =* 31), renal (*n =* 24), neuroendocrine (*n =* 12), adrenal (*n =* 9), germ-cell (*n =* 7), pancreatic (*n =* 4), thymic (*n =* 4), IMT (*n =* 4), soft tissue (*n =* 5)	Pheochromocytoma (*n =* 6), paraganglioma (*n =* 6), adrenocortical adenoma (*n =* 1), bilateral carney complex (*n =* 2), bilateral McCune–Albright (*n =* 2), mature teratoma (*n =* 2), malignant seminoma (*n =* 1), non-seminomatous (*n =* 4), neuroendocrine tumor (*n =* 1), focal hyperinsulinism (*n =* 3), thymoma (*n =* 4), myasthenia (*n =* 1), MEN1 (*n =* 1), IMTs (*n =* 4), embryonal rhabdomyosarcoma (*n =* 1), neurofibroma (*n =* 1), bronchial carcinoid tumor (*n =* 1), leiomyoma (*n =* 1), lipoma (*n =* 1)	3:5.6	92	8.2 (3.6–13)	26 (15–47)	215 (156–282)	57 (*n =* 51)	8 (*n =* 7)	8 (*n =* 7)	Renal vein injury in Wilms' tumor (*n =* 1), misdiagnosed renal vein tumor thrombus and spillage (*n =* 1), poor respiratory tolerance after diaphragmatic resection and spillage due to tumor rupture after the conversion in WT infiltrating the liver (*n =* 1), Sliding Hem-O-Lock clip-on renal vein (*n =* 1), difficult renal hilum dissection in renal sarcoma (*n =* 1), difficult dissection in a neuroblastoma and ganglioneuroma for narrow space and vascular involvement (*n =* 2)	5.7 (*n =* 5)	III (*n =* 4), II (*n =* 1)	Pneumothorax (*n =* 2), anastomotic stenosis (*n =* 1), adhesions (*n =* 1), retroperitoneal collection (*n =* 1)	3	27 (18–29)	2% (*n =* 2)
Li et al. (12)	2021	8	Bladder/prostate tumor (*n =* 8)	Bladder rhabdomyosarcoma (*n =* 8)	5:03	8	6 (0.9–11)	NA	172 (104–316)	100 (*n =* 8)	0	0		0	/		12.5	13.3	0

*LHS, length of hospital stay*.

The intraoperative complication rate ranged from 0 to 28%, and the main reported causes were difficult dissection and intraoperative discovery of more invading tumors than expected. Moreover, two conversions were performed due to a lack of confidence in the anatomy. No robotics-related complication was reported (e.g., injury to the patients due to robotic arms).

The median operative time, including docking, was 184 min. The postoperative complication rate accounted for 4% (most reported complications were pneumothorax, unexpected drug reaction, and adhesions). The median hospital stay was 4.6 days.

Follow-up, when stated, was carried out for a median of 14.4 months. The recurrence rate was 1.4%.

A comparison between our experience and the literature is reported in Table 3.

**Table 3:** Comparison between our experience and literature.

	**Our cohort**	**Literature**
Patients (*n*)	14	134
M:F	0:1	1:2
Age at surgery (years)	11.5 (5.4–16.9)	9 (0.9–19)
Malignancy rate	14%	65%
Operative time (min)	166	184
Intraoperative complication rate	14.2% (2 spillage)	4.5% (0–28%)
Conversion rate	0%	5%
Recurrence rate	0%	3.20%

## Discussion

The role of RAS is becoming progressively more important in every field, including pediatric oncology. In the last decade, several papers have been published on the subjects, even if the sample of the cohort is often small since most of the studies are case reports (5–12).

The still limited spread of this technique is due to both the concerns of the use of MIS for tumors and the well-known limitations of RAS in children (13, 14). Nevertheless, since the first cases reported by Meehan and Sandler (15), results have been encouraging.

To date, the published paper underlines the necessity to perform a strict selection of all children undergoing RAS, in order to adhere to oncological guidelines. The application of this technique requires an in-depth knowledge of pediatric oncology and the revision of each case by an ongoing multidisciplinary team, composed of medical oncologists, radiologists, anesthesiologists, and surgeons experienced in MIS and oncologic surgery (16).

Comparing our experience with the literature (Table 3), we found a different M:F ratio (0:1 vs. 1:2), probably due to our initial selection to perform surgery in adnexal lesions. In our practice, the first oncological procedures that were performed concerned adnexal lesions, as we believed that RAS is a perfect fit for these indications. Adult gynecology has already proven the feasibility of robotic procedures for both benign and malign pathologies (7, 17–20), and experience in pediatrics is growing (7, 9, 21). A robot allows a superb visualization of the pelvis, and in the majority of cases, port placement may be carried out easily, as most girls undergoing this kind of surgery are adolescents. Alongside, MIS offers good cosmetic results, which is an important factor, especially in this group of patients (22). Nevertheless, the relative simplicity of the surgical procedure must not let the surgeons underestimate the risk of spillage and/or rupture of ovarian lesions.

Although extremely rare, malignant ovarian neoplasm in children and adolescents may occur (23–27). If preoperative examinations point out the risk of malignancy, oophorectomy should be strongly considered, and, when performed, no salpingectomy is required, which is preferable in this age group (24, 25, 28, 29). Nevertheless, in pediatrics, there is an interest in preserving as much ovarian tissue as possible, to assure the development of normal puberty and future fertility (30). As many articles describe how laparoscopy may be safely applied to perform ovarian-sparing surgery in pediatrics, this topic is sometimes debated (31). In our opinion, the already cited technological advantages of the da Vinci system may further allow a surgeon to perform a safe excision minimizing the risk of spillage, as long as all oncological principles are followed (e.g., preoperative tumor markers, adequate imaging, and extraction of the mass using an Endobag).

Risks of tumor rupture and/or spillage, risk of incomplete resection, and risk of port-site recurrences count as the most cited problems for MIS/RAS.

In our experience, the complication rate was higher than in other series (14.2%, two spillages, vs. 4.5%). Spillage during RAS is reported in only one case by Blanc et al. (11), due to the leakage of a renal vein thrombus of a Wilms tumor, discovered after renal vein control. Overall, the spillage rate was 0.7%. Despite that the risk should not be underestimated, the use of MIS in malignancies where spillage or rupture is particularly dangerous has been accepted in selected cases. For example, in 2014, the Renal Tumor Strategy Group of the International Society of Paediatric Oncology (SIOP) published the largest cohort of laparoscopic excision of Wilms tumor (32). Moreover, in the same year, the SIOP Umbrella protocol (33, 34) proposed inclusion criteria to safely perform laparoscopic nephrectomy in Wilms tumors. Finally, in 2018, Bouty et al. showed, by performing a systematic review, that in highly selected cases, MIS in Wilms tumor did not worsen prognosis (35).

Although detractors of RAS are skeptical about its use due to the absence of haptic feedback, the technological advantages of the da Vinci system (3D vision, seven degrees of freedom, tremor filtration, and precise camera control) have expanded the possibilities of performing and reproducing difficult operations, especially when there is a deep and narrow field and when fine dissection is required for delicate tissue manipulation, as is the case in pediatric oncology surgery (16, 36).

Regarding the suspected incidence of port-site recurrences, a recent publication in adults shows equivalent outcomes between laparoscopic/robotic and open approaches (17, 37). In pediatrics, no recurrences have been cited so far.

In literature, the overall conversion rate to open surgery was about 5%, and difficult dissection or surgeon diffidence in continuing RAS were the most reported causes.

Conversion is required every time there is the possibility to upstage the tumor. Nevertheless, as the experience of the surgeon grows, a reduction in the rate of conversion is reported (11).

This is certainly due to improved confidence in RAS, associated with a better selection of patients addressed to this technique. Blanc et al. suggest beginning the experience with RAS with smaller tumors and converting in cases of difficult dissection, stressing that the main objective is to respect the oncological surgical principles (11).

For several authors, surgeons with or without previous laparoscopic or robotic experience could perform independently and properly robotic procedures (38, 39). In surgical oncology, the passage from open to laparoscopy or RAS is far from being easy. The approach to pediatric tumors needs an important surgical background that comes from open surgery. To apply RAS in tumor resection, it is not only necessary to improve personal learning curve, training, and exercising on virtual and animal models mastering basic and advanced robotic skills. For any surgeon, it is necessary to perform at least 250 procedures to consider himself/herself independent and a mentor in surgery.

The availability of senior surgeons with experience in both oncologic surgery and MIS provides valid support to the steep learning curve.

In our experience, the availability of a simulating station for the da Vinci system allowed us to perform specific personal training, both virtual and *in vivo*. The approach to pediatric tumors came after a consistent experience in other RAS and specific training of the whole surgical team. Thanks to the presence of 2 consoles, it was possible for younger surgeons to approach tumors, with senior surgeons mentoring live, even those with less experience in RAS.

An oncological procedure carried out with RAS, especially at the beginning, may require a long operative time. Installation of the patient requires meticulous attention. Comfortable positioning as well as the use of adequate padding and skin protection must always be verified (4, 40). It is important to avoid hyperextension or flexion in small children, as they are more pliable compared with adolescents and adults (4). Once the patient is correctly installed, the docking procedure needs to be correctly planned, especially in infants and toddlers, as the working space is limited. Particular attention is needed to avoid conflict between the robotic arms and, more importantly, between the robot and the patient. The key role in assuring and controlling potential harm to the child during surgery is played by the scrubbed nurse and the scrubbed assistant who need to control and verify patient safety throughout the intervention, alongside assisting the lead surgeon by passing needles, bandages, or other instruments through the assistant port (41).

In our experience, OT was comparable with that in the literature (166 vs. 184 min).

Anesthesiologists involved in RAS procedures must be familiar with the robot and its installation, as well as the degree of movements of the arms. All vascular accesses must be positioned before docking and arranged to minimize any possible conflicts with the robot. At the same time, robot installation must not prevent the work of the anesthesiology team during surgery.

In literature, concerning pediatric oncology, no case of robotics-related complication has been reported, in terms of injury to the patient due to a robotic arm, nor cases of robot malfunction. When operating with the da Vinci robot, especially in the case of delicate surgeries such as oncological procedures, all members of the surgical team have to keep in mind the possibility of malfunction and must be able to respond and properly provide assistance if necessary. In fact, during any robotics-assisted procedure, the role of the technical assistance team is crucial. Technical support should always be available and consists of in-person and phone support provided (42). Their help can solve most cases of dysfunction of the robot or any of its components. In our experience, we were assisted by a da Vinci specialist during the most complicated surgeries.

## Limitations

The limitations of this study are represented by the retrospective nature of the analysis and the small cohort of patients (14) with a high prevalence of adnexal lesions. Since the application of RAS to pediatric oncology represents a new experience, even the systematic review is limited by a low number of papers with a small series.

## Conclusions

RAS in pediatric oncology has proven to be feasible for different pathologies. Although optimistic reports have been published in the literature, the use of RAS should be limited to selected cases and performed by highly trained oncological surgeons. So far, the literature strongly recommends the presence of a multidisciplinary board of experts (surgeon, anesthesiologist, radiologist, and oncologist) to evaluate candidates to RAS. All procedures must be carried out while respecting oncological protocols. Preparation and patient positioning, alongside a correct port placement, are crucial to safely perform these surgeries.

Further studies are needed to assess the role of RAS in pediatric oncology, as well as to implement specific technical standards for each pediatric tumor.

## Data Availability Statement

The original contributions presented in the study are included in the article/supplementary material, further inquiries can be directed to the corresponding author/s.

## Author Contributions

GR contributed to conception and design of the study. FV and MG organized the database and performed the statistical analysis. FV, MB, and AR wrote the first draft of the manuscript. FV, MB, and GR wrote sections of the manuscript. LA and RV reviewed the manuscript. All authors contributed to manuscript revision, read, and approved the submitted version.

## Conflict of Interest

The authors declare that the research was conducted in the absence of any commercial or financial relationships that could be construed as a potential conflict of interest.

## Publisher's Note

All claims expressed in this article are solely those of the authors and do not necessarily represent those of their affiliated organizations, or those of the publisher, the editors and the reviewers. Any product that may be evaluated in this article, or claim that may be made by its manufacturer, is not guaranteed or endorsed by the publisher.
